# Impact of aging on food consumption in rural China: Implications for dietary upgrading and health improvement

**DOI:** 10.3389/fnut.2022.933343

**Published:** 2022-11-24

**Authors:** Ming Gao, Bi Wu, Wencheng Jin, Jiashuo Wei, Jiwen Wang, Jinkai Li

**Affiliations:** ^1^Research Center for Rural Economy, Ministry of Agriculture and Rural Affairs, Beijing, China; ^2^Institute of Rural Development, Chinese Academy of Social Sciences, Beijing, China; ^3^National Agricultural and Rural Development Research Institute, China Agricultural University, Beijing, China; ^4^Department of Economics, Faculty of Economics and Business Administration, Ghent University, Ghent, Belgium

**Keywords:** aging, food consumption, intergenerational cohabitation, household composition, health improvement

## Abstract

**Background:**

The issue of population aging in rural China is getting profound; nevertheless, its impact on food consumption has not been well evaluated. This study aims to examine the relationship between rural aging and family food consumption in rural China.

**Materials and methods:**

Using the statistical yearbook data and the nationally representative household-level data from the China Rural Fixed Observation Points, this study compares the evolution of food consumption between rural and urban residents from 1985 to 2020 and analyzes the structure of food consumption expenditure of rural residents. Next, this study further investigates the impact of aging on food consumption in rural households with ordinary least squares.

**Results:**

(1) The principal foods consumed by rural residents in 2020 are meat and meat products (36.8%), grain (24.5%), and vegetables (10.9%). (2) An increase in older adults has decreased the absolute consumption of all foods, while it increased relative consumption of meat and meat products, aquatic products, edible oil and fats, poultry, eggs, and sugar. (3) Due to differences in the structure of young adults’ food consumption, older adults would increase their consumption of fruits and vegetables if they lived with younger adults.

**Conclusion:**

The findings of this study suggest that rural older adults may increase their consumption of fruits and vegetables by advocating intergenerational cohabitation while maintaining their intake of protein to achieve a balanced dietary structure and improve their health condition.

## Introduction

Reasonable food consumption and nutrition intake are basic guarantees of physical health and high-quality human capital, which are of great importance to increasing labor productivity and promoting long-term social development (1). Combined with steady growth in income level, the total food consumption of Chinese residents has significantly increased (2). In contrast to food consumption in China in 1978, the consumption of cereals, meat, dairy products, and vegetables and fruits in 2015 reached 455.2, 91.8, 51.1, and 553 million tons, respectively, which represented increases of 220, 730, 1,610, and 950%, respectively (3). Rural residents have a lower intake of protein and vitamins and an excessive intake of staple foods compared with urban residents (4). By the end of 2020, there were still 510 million rural residents, accounting for 36.11% of the total population of China (5), indicating that the issue of food consumption by rural residents cannot be ignored.

Meanwhile, China is facing severe aging challenges; according to the Seventh National Census in 2020, the number of people aged over 60 years reached 264 million (18.70%). The issue of aging in rural areas is even more severe, and the proportion of older adults in rural areas is 23.81%, which is much higher than that in urban areas (6). The rapid increase in the proportion of older adults in rural China has profoundly impacted rural residents’ food consumption (7). Noticeably, as aging continues to be a serious problem in rural areas (8), the rise in the proportion of older adults may have been an increasingly important factor that affects food consumption in rural China.

Generally, there are usually two types of influencing factors that affect food consumption: macrolevel factors, such as agricultural production, economic growth, urbanization, and infrastructure construction (9–12); and microlevel factors, such as education level, household income, family structure, food prices, and social and psychological traits (2, 13–16). There is also a small section of the literature that focuses on the impact of aging on food consumption. Considering the health condition of older adults, less physical activity and degenerated body organs usually lead to a decline in total food consumption (17, 18). There is empirical evidence that accelerated aging may decrease food consumption per capita (19). However, a decrease in total food consumption does not mean a decrease in each type of food consumption. Although rural older adults choose to decrease meat consumption to some extent (20), they also consciously prefer to consume healthier foods to obtain more nutrients (21–23). In addition, older adults’ gender, income level, personal traits (24), and family structure are also important factors affecting food consumption (25–29).

From the above literature review, the following gaps require more attention. First, most of the aforementioned literature only considers the impact of aging at the regional level, which may lack the analysis of national micro household survey data. Second, there seems to be less research on the impact of aging on food consumption by rural residents, with most related studies paying attention to Chinese residents or urban residents. Finally, there is little research on how aging in rural China affects specific food consumption at the household level. Most available studies pay more attention to the consumption of staple foods and meat, ignoring aquatic products, dairy products, fruits, and vegetables. However, these types of foods are also an important part of rural residents’ food consumption.

This study aims to explain how aging affects family food consumption structures and expenditures in rural families, thereby providing empirical evidence and policy implications for the improvement of diet and health of rural residents. Additionally, this study seeks to fill various research gaps. We use nationally representative household survey data to study the impacts of older adults on food consumption, which makes the conclusions more reliable. We divide food consumption into 10 categories, which helps us understand the impact of aging on specific food consumption more accurately. This study also considers the impact of young and older adults living together, which complements the analysis of the external effect of demographic shifts on households. Considering older adults’ low vitamin intake, this study puts forward the beneficial countermeasures that older adults should increase expenditure on fruits and vegetables and maintain the intake of protein to improve their dietary structure and enhance physical fitness.

## Materials and methods

### Data

In our study, we use two types of data. The first type of data is from the National Household Income and Expenditure Survey, which is carried out by the National Bureau of Statistics of the Chinese Government. National Household Income and Expenditure Survey adopted a stratified multi-stage random sampling method and included 160,000 urban and rural households from 31 provinces of China. The survey includes detailed records about household level food consumption. The National Bureau of Statistics aggregated household level data, conducted weighted statistics, and then released the annual report about the average food consumption of urban and rural residents. Notably, the micro household data are not publicly available, and we could only obtain information about the aggregated average food consumption of urban and rural residents from the annual report. More specifically, four types of food consumption per capita of urban and rural residents from 1985 to 2020 are used for the analysis of rural–urban residents’ food consumption in section “The evolution of rural-urban residents’ food consumption”.

The second type of data used in this study was retrieved from the China Rural Fixed Observation Points in 2020, collected by the Research Center for Rural Economy under the Ministry of Agricultural and Rural Affairs of China, which includes comprehensive information about rural households and their members. This survey data only target rural households, not urban households, which is used to analyze the structure of food consumption of rural households in section “The structure of food consumption expenditure in rural households” and the regressions on the effects of rural aging on food consumption in sections “Regression of food consumption per capita on aging” and “Regression of food consumption structure on aging”. The survey was formally set up in 1986 and has been run until now, covering 23,000 rural households and 375 villages in 368 counties of 31 provinces. It is nationally representative survey data. Equal survey weight is given to poor, medium, and rich rural families in typical counties of each province in China. These data have been widely used in academic research (30–33). The data consist of 1,250 indicators in eight categories, including labor force, annual household income, and consumption. Notably, detailed information regarding the consumption of various foods by rural households is available in 2020, which builds a solid foundation for this research. Thus, we only use survey data from 2020, and the final sample size for the empirical analysis was 20,185 after removing observations with missing values.

### Measurement

We focused on two dependent variables: (1) the food consumption expenditure per capita on a specific food (unit: yuan) and (2) the proportion of the consumption expenditure on a specific food in total expenditure (unit:%). These two variables reflected the quantity and structure of food consumption among rural residents. Notably, we obtained information on family food expenditures and family members from the Household Questionnaire. The Household Questionnaire is generally answered by the head of the household. Heads of households usually have accurate information about family members and food consumption and provide clear answers, guaranteeing high-quality data. Thus, per capita expenditure is accurate. The detailed food items can be found in Supplementary Table 1.

Based on the classification of food consumption by the National Bureau of Statistics of China, 10 main types of food were selected: (1) grains, (2) edible oil and fats, (3) vegetables and edible mushrooms, (4) meat and meat products, (5) poultry, (6) aquatic products, (7) eggs, (8) milk and dairy products, (9) melons and fruits, and (10) sugar.

Additionally, we focused on two core independent variables: (1) the number of people in the household aged 65 years and over (NP65) and (2) the proportion of people aged 65 years and over (PP65). These two independent variables were treated as follows: (1) NP65 was transformed into a categorical variable with values of 0, 1, and 2, where 2 indicates the number of older adults equal to or more than 2. There were 57.1% of rural households with no older adults, 21.4% of rural households with one older adult, and 21.5% of rural households with two or more older adults. (2) For PP65, we defined three types of rural households: pure young family (PP65 = 0), mixed family (0 < PP65 < 1), and pure older adult family (PP65 = 1). The proportions of pure young, mixed, and pure older adult families were 57.1, 13.4, and 29.4%, respectively.

We excluded the confounding influence of wealth (34), workforce composition (35), and other demographic characteristics at the household level by adding the following controlling variables: mean age of household members weighted by the length of stay at home, which is used for controlling for the interference of family member’s age; whether the household was poor, and the proportion of poor families was 7.4%, which is used for controlling family wealth status; annual household income because household income level directly determines the quantity and quality of food consumption (36); and the proportion of labor force, which may affect family food consumption patterns (37). The descriptive statistics of relative variables can be seen in Table 1.

**TABLE 1:** Descriptive statistics of continuous variables (*N* = 20,185).

Variables	Mean	Std. dev.
Per capita consumption expenditure of grain (yuan)	24.5	14.76
Per capita consumption expenditure of edible oil and fats (yuan)	9.0	6.15
Per capita consumption expenditure of vegetables and edible mushrooms (yuan)	10.9	8.53
Per capita consumption expenditure of meat and meat products (yuan)	36.8	15.02
Per capita consumption expenditure of poultry (yuan)	3.3	4.09
Per capita consumption expenditure of aquatic products (yuan)	4.3	4.73
Per capita consumption expenditure of eggs (yuan)	3.2	3.14
Per capita consumption expenditure of milk and dairy products (yuan)	2.7	4.11
Per capita consumption expenditure of melons and fruits (yuan)	4.7	4.41
Per capita consumption expenditure of sugar (yuan)	0.7	0.83
Weighted mean age (years)	39.7	18.64
Household income (10,000 yuan)	8.4	7.04
Proportion of labor force (%)	65.0	30.38

1 RMB (Yuan) = 0.14493 USD (dollar) in 2020.

### Analytical strategy

This study’s analysis was conducted in two parts. First, we performed a descriptive statistical analysis of rural and urban residents’ food consumption evolution with the aggregated data of the National Household Income and Expenditure Survey, which aimed to identify the consumption variation in four main food types of rural and urban residents. Due to the data availability, we cannot conduct statistical tests with household data, but only conduct descriptive statistics with aggregated data. We also describe the composition of food consumption expenditure in rural households with the micro data of China Rural Fixed Observation Points in 2020 in section “The structure of food consumption expenditure in rural households”.

Second, we used ordinary least squares (OLS) to empirically analyze the relationship between aging and food consumption in rural households (38) with the micro data of China Rural Fixed Observation Points in 2020. There are four analyses: the relationship between the number of older adults and the food consumption per capita, the relationship between household composition and food consumption per capita, the relationship between the number of older adults and the structure of food consumption, and the relationship between household composition and the structure of food consumption.

## Results

### Trends and composition of food consumption

In this section, we first analyze the evolution of food consumption of grains, pork, aquatic products, and edible vegetable oil separately from 1985 to 2020 among rural and urban residents; and second, we concentrate on the structure of food consumption expenditure of rural residents in detail with the latest data from unique nationally representative household survey data.

#### The evolution of rural–urban residents’ food consumption

**FIGURE 1:**
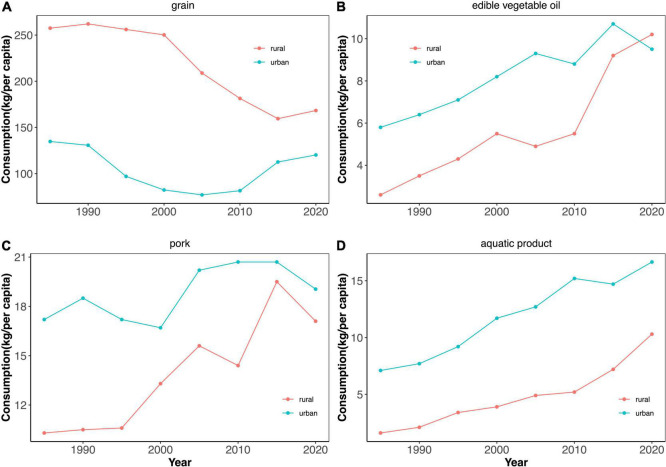
Urban-rural trends of per capita consumption of four main foods from 1985 to 2020. The data has been aggregated to the urban and rural average level from the micro household level by the National Bureau of Statistics. The occasion of observation is equal to 36 (More specifically, 36 for the average food consumption of rural residents from 1985 to 2020, and 36 for the average food consumption of urban residents in each year from 1985 to 2020). The red and blue lines denote per capita consumption of grain, edible vegetable oil, pork, and aquatic product of rural and urban residents, respectively.

From Figure 1A, the grain consumption gap between urban and rural residents has narrowed continuously, from 122.7 kg per capita in 1985 to 48.2 kg per capita in 2020. However, there is still a noticeable gap of 48.2 kg per capita.

From Figure 1B, Chinese residents’ edible vegetable oil consumption shows a trend of fluctuating growth. Usually, rural residents’ edible vegetable oil consumption is significantly lower than that of urban residents. However, this was the first time that the consumption of edible vegetable oil by rural residents surpassed that of urban residents in 2018.

From Figure 1C, the pork consumption of rural residents caught up with that of urban residents from 1985 to 2020. Noticeably, in 2018, rural residents’ pork consumption (23 kg per capita) surpassed urban residents’ pork consumption (22.7 kg per capita), showing the rapidly growing demand of rural residents for pork. Simultaneously, the pork consumption of rural and urban residents seems to have peaked at 20 kg per capita.

From Figure 1D, the strong growth in aquatic product consumption is indicated by an increase of 235.21% and 643.75% for urban and rural residents, respectively. In the 1980s, the aquatic product consumption of urban and rural residents was 7.1 and 1.6 kg per capita, respectively. In recent years, the growth rate of aquatic products for rural residents has been much higher than that of urban residents. Although the urban–rural gap in aquatic product consumption has greatly decreased over the past 35 years, there is still a disparity.

#### The structure of food consumption expenditure in rural households

**FIGURE 2:**
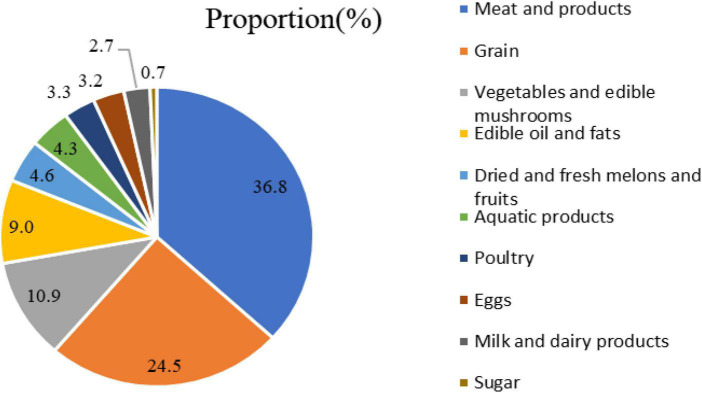
Rural residents’ food consumption expenditure structure in 2020, unit: %.

We also discuss the latest rural households’ food consumption expenditures using survey data from 2020. In Figure 2, there are 10 main types of food in the rural residents’ food list. Rural residents’ food consumption structure has gradually transformed from a single to a diversified structure. From the perspective of food composition, meat and meat product consumption was ranked first, accounting for more than one-third of total food expenses. The proportion of grain consumption was 24.52% and ranked second. The expenditure on vegetables and edible mushrooms approximately equals that of edible oil and fats, accounting for 10% of the total cost. However, aquatic product, egg, milk, and dairy product expenditures still represent a small proportion, adding up to only 10%, indicating that rural residents’ protein intake may be insufficient and the quality of food consumption needs further improvement.

### Regression of food consumption per capita on aging

From Tables 2, 3, we find that food consumption per capita is related to the number and proportion of older adults in rural households.

**TABLE 2:** Regression of the food consumption per capita on the number of older adults.

	(1)	(2)	(3)	(4)	(5)	(6)	(7)	(8)	(9)	(10)
Variables	Grain	Edible oil and fats	Vegetables and edible mushrooms	Meat and meat products	Poultry	Aquatic products	Eggs	Milk and dairy products	Melons and fruits	Sugar
**Zero old = ref.**										
one old	−53.76***	−10.43***	−31.71***	−67.09***	−5.686**	−9.979***	−5.843***	−4.689***	−21.93***	−1.248***
	(6.069)	(2.419)	(5.159)	(11.78)	(2.255)	(2.604)	(1.228)	(1.615)	(2.070)	(0.252)
two old	−108.6***	−34.64***	−86.77***	−177.8***	−17.59***	−21.62***	−13.22***	−12.83***	−30.61***	−3.016***
	(6.668)	(2.658)	(5.669)	(12.94)	(2.478)	(2.862)	(1.349)	(1.774)	(2.274)	(0.277)
R-squared	0.051	0.036	0.036	0.040	0.027	0.057	0.038	0.028	0.044	0.035

**p < 0.05; ***p < 0.01. *N* = 20,185. Covariates include weighted mean age, household income, poverty status, and proportion of labor force (%).

**TABLE 3:** Regression of the food consumption per capita on the household composition.

	(1)	(2)	(3)	(4)	(5)	(6)	(7)	(8)	(9)	(10)
Variables	Grain	Edible oil and fats	Vegetables and edible mushrooms	Meat and meat products	Poultry	Aquatic products	Eggs	Milk and dairy products	Melons and fruits	Sugar
**Young household = ref.**										
Mixed household	−99.30***	−27.15***	−65.32***	−140.6***	−15.88***	−22.27***	−13.58***	−11.68***	−29.14***	−2.467***
	(5.436)	(2.175)	(4.649)	(10.60)	(2.027)	(2.337)	(1.099)	(1.452)	(1.863)	(0.227)
Old household	48.91***	15.88***	3.455	36.89**	18.32***	26.49***	16.98***	11.83***	−5.726*	0.668*
	(8.888)	(3.556)	(7.601)	(17.34)	(3.315)	(3.822)	(1.796)	(2.374)	(3.046)	(0.372)
R-squared	0.062	0.040	0.037	0.042	0.032	0.065	0.051	0.033	0.047	0.037

*p < 0.1; **p < 0.05; ***p < 0.01. *N* = 20,185. Covariates include weighted mean age, household income, poverty status, and proportion of labor force (%).

#### Regression of food consumption per capita on the number of older adults

Table 2 indicates that an increase in the number of older adults significantly lowers the consumption of all 10 types of food. With the increase in older adults, the decline in spending on meat and meat products and grains ranked first and second, respectively. Compared with the family with no older adults, the consumption of meat and meat products and grain in the family with one older adult decreased by 67 and 54 yuan, respectively. In the case of a family with two or more older adults, the decrease in meat and meat products and grains was 178 and 109 yuan, respectively. Compared with the family with no older adults, spending on vegetables and edible mushrooms, edible oil and fats, and melons and fruits in the family with one older adult decreased by 32, 10, and 22 yuan, respectively. However, the spending on these three types of food for those families with two or more older adults decreases by 87, 35, and 31 yuan, respectively. In addition, it is worth noting that there is a smaller decrease in the consumption of aquatic products, poultry, eggs, milk and dairy products, and sugar.

#### Regression of food consumption per capita on household composition

Table 3 shows that the food consumption of mixed families is significantly lower than that of young families. Specifically, food consumption per capita on meat and meat products and grains in mixed families is 140 and 99 yuan lower than in young families, which is the main difference between these two types of families. The gaps in vegetables and edible mushrooms, edible oil and fats, and melons and fruits are 65, 27, and 29 yuan, respectively. Furthermore, the gaps in poultry, aquatic products, eggs, milk and dairy products, and sugar are relatively small, at no more than 25 yuan. Noticeably, food consumption per capita in pure older adult families is significantly higher than that in young families. The most obvious gaps between young and older adult families are reflected in the consumption of grain and meat and meat products, at 49 and 37 yuan, respectively.

### Regression of food consumption structure on aging

Next, we analyze the impact of the number and proportion of older adults on the structure of food consumption at the household level.

#### Regression of food consumption structure on the number of older adults

Table 4 shows the regression results of the proportion of the consumption of various foods on the number of older adults. With an increase in the number of older adults, the variation in different types of foods is the opposite. First, the proportion of grains, vegetables, and edible mushrooms and melons and fruits declined, which is consistent with the variation in corresponding food consumption. Compared with a family with no older adults, having one or two older adults in the family reduces the proportion of grain consumption by 1.0 or 1.2 percentage points, while the decrease was 0.1 and 0.8 percentage points for vegetables and edible mushrooms, respectively, and 0.5 and 0.4 percentage points for melons and fruits, respectively. Second, the proportion of consumption of edible oil and fats, meat and meat products, poultry, aquatic products, eggs, milk and dairy products, and sugar increased, even though there are more older adults in the household. Among these types of food, the proportion of meat and meat product consumption increased the most by 1 and 1.3 percentage points, respectively. In addition, in a family with two older adults, the increase in the proportion of food consumption of edible oil and fats, poultry, aquatic products, eggs, and sugar was 0.25, 0.2, 0.5, 0.1, and 0.04 percentage points, respectively.

**TABLE 4:** Regression of the proportion of food consumption (%) on the number of older adults.

	(1)	(2)	(3)	(4)	(5)	(6)	(7)	(8)	(9)	(10)
Variables	Grain	Edible oil and fats	Vegetables and edible mushrooms	Meat and meat products	Poultry	Aquatic products	Eggs	Milk and dairy products	Melons and fruits	Sugar
**Zero old = ref.**										
one old	−0.972***	0.333***	−0.110	0.979***	0.0990	0.0805	−0.0364	0.109	−0.496***	0.0153
	(0.281)	(0.117)	(0.162)	(0.287)	(0.0777)	(0.0889)	(0.0600)	(0.0785)	(0.0840)	(0.0157)
two old	−1.204***	0.249*	−0.827***	1.255***	0.171**	0.447***	0.131**	0.134	−0.394***	0.0391**
	(0.309)	(0.129)	(0.179)	(0.316)	(0.0854)	(0.0977)	(0.0660)	(0.0863)	(0.0923)	(0.0173)
R-squared	0.015	0.011	0.014	0.006	0.020	0.040	0.007	0.008	0.014	0.011

*p < 0.1; **p < 0.05; ***p < 0.01. *N* = 20,185. Covariates include weighted mean age, household income, poverty status, and proportion of labor force (%).

#### Regression of food consumption structure on household composition

From the perspective of family types (such as young, mixed, and older adult families), we can see the structure of food consumption from Table 5. Based on the results in this table, we classify the impact of the proportion of older adults into three categories. First, for vegetables and edible mushrooms, and melons and fruits, the proportion of consumption of vegetables and edible mushrooms in mixed and older adult families is 0.3 and 0.8 percentage points lower than that in young families, and the proportion of spending on melons and fruits is 0.4 and 0.8 percentage points lower. Thus, due to differences in the structure of young adults’ food consumption, older adults would increase their consumption of fruits and vegetables if they lived with younger adults.

**TABLE 5:** Regression of the proportion of food consumption (%) on the household composition.

	(1)	(2)	(3)	(4)	(5)	(6)	(7)	(8)	(9)	(10)
Variables	Grain	Edible oil and fats	Vegetables and edible mushrooms	Meat and meat products	Poultry	Aquatic products	Eggs	Milk and dairy products	Melons and fruits	Sugar
**Young household = ref.**										
Mixed household	1.287***	0.340***	−0.339**	1.379***	0.0922	0.155*	−0.0513	0.0731	−0.393***	0.0311**
	(0.253)	(0.106)	(0.146)	(0.259)	(0.0700)	(0.0801)	(0.0541)	(0.0708)	(0.0757)	(0.0142)
Old household	0.144	0.0616	−0.822***	−0.493	0.336***	0.682***	0.512***	0.378***	−0.791***	−0.00671
	(0.414)	(0.173)	(0.239)	(0.423)	(0.115)	(0.131)	(0.0884)	(0.116)	(0.124)	(0.0232)
R-squared	0.016	0.011	0.013	0.007	0.020	0.040	0.009	0.008	0.015	0.011

*p < 0.1; **p < 0.05; ***p < 0.01. *N* = 20,185. Covariates include weighted mean age, household income, poverty status, and proportion of labor force (%).

Second, there were significant differences in the proportion of consumption of poultry, aquatic products, eggs, and milk and dairy products between older adults and young families. Compared with young families, the proportion of older adult families’ consumption of these four types of food is 0.3, 0.7, 0.5, and 0.4 percentage points lower, respectively.

Third, there are observable differences in the consumption of grain, edible oil and fats, meat and meat products, and sugar between mixed and young families. The proportion of food consumption of these types of food in the mixed families was 1.3, 0.3, 1.4, and 0.03 percentage points higher than that in the young families.

## Discussion and conclusion

This study described the food consumption trends of rural and urban residents from 1985 to 2020 and the structure of food consumption of rural residents in China in 2020. Since 1985, grain consumption per capita in rural areas has decreased gradually. However, there is still one noticeable difference in grain consumption between rural and urban residents (4). The growth rate of aquatic product consumption among rural residents was faster than that among urban residents. However, the consumption of edible oil and fats, and pork per capita in rural areas was equal to that in urban areas, which shows the huge demand for meat and meat products and oil in rural China. By 2020, the proportion of food consumption of both meat and meat products and grain in the total food consumption accounted for 61%. This result shows that rural residents’ food consumption tends to be diverse (39). Along with the increase in the supply of various types of food, rural residents’ demand for high-quality food has been satisfied to some extent (40).

Next, we studied the relationship between aging and food consumption in rural China. In 2020, Engel’s coefficient of rural residents was approximately 32.7%, which is not in line with the actual situation in rural residents’ living levels (41). Thus, we pay attention not only to how much food rural residents consume but also to what kind of food they consume. At the household level, the presence of older adults may significantly affect the structure of food consumption. This study focuses on the impact of the number of older adults and household composition on the amount and structure of food consumption at the household level.

On one hand, an increase in the number of older adults improves relative food consumption of edible oil and fats, poultry, aquatic products, eggs, milk and dairy products, and sugar. In addition, food consumption per capita would increase if all family members were older adults. This may be related to the amount of time that older adults spend at home. Generally, older adults spend more time at home, whereas young adults spend less time there (42). Therefore, the food consumption per capita in pure older adult families is greater than that in pure young families. As mixed families are usually made up of three generations and children usually have lunch at school, food consumption per capita in mixed families is the lowest. Noticeably, there is an interesting phenomenon that, compared with young people, older adults consume less of melons and fruits, which is consistent with the previous literature (43). Thus, the fruit consumption of both pure older adult families and mixed families is lower than that of pure young families. This suggests that older adults have a lower preference for fruit. Although such products may be unregistered in rural areas due to their own production and consumption, rural residents’ consumption of fruit and vegetables is still relatively low compared with urban residents’ consumption (44).

On the other hand, we found empirical evidence that intergenerational cohabitation may affect the structure of food consumption. Importantly, older adults increase their consumption of fruits and vegetables if they live with younger adults. There are obvious similarities and differences in the structure of food consumption between older and younger people (45). First, regarding the proportion of total consumption of grain, edible oil and fats, meat and meat products, and sugar, older adults and younger people show similar food consumption structures. However, when older adults and young people live together, the aforementioned food consumption structure shows variations with different characteristics. Second, there are significant differences in food consumption between young and older adults in terms of poultry, aquatic products, eggs, milk and dairy products, vegetables and edible mushrooms, and melons and fruits (46). When they live together, the differences become narrow or even disappear.

This study may contribute to the following aspects. First, we find that the number of older adults and household composition significantly affect the amount and structure of food consumption in Chinese rural households. Second, this study focuses on analyzing the impact of intergenerational cohabitation, such as older adults and young people living together on the structure of food consumption. Specifically, this study complements the analysis of the second demographic transition in China and concludes that there are pronounced differences in food consumption among various family types, such as in pure older adult, mixed, and pure young families.

This study draws the following main conclusions. First, since the reform and opening up, food consumption of Chinese rural residents has tended to be diversified, consistent with previous studies (39). Second, this study provides empirical evidence that aging significantly affects the food consumption of rural residents. More specifically, food consumption per capita is related to the number of older adults and household composition in rural households. Third, older and younger family members show different preferences regarding food consumption, and intergenerational cohabitation may significantly affect the structure of family food consumption.

The conclusions of this study could provide a decision-making basis for promoting food consumption diversification and improving the quality of food consumption by older adults in rural China. To ensure the balance of dietary structure and health improvement of rural older adults, we suggest that rural older adults should increase their consumption of fruits and vegetables by advocating intergenerational cohabitation and maintain a sufficient intake of protein by appropriately increasing the consumption of high-protein beef and fish.

It is worth noting that this study may have the following limitations due to the restriction of data. First, we compare the food consumption of the rural and urban residents as a background, while it is not feasible to conduct statistical tests for the food consumption of rural and urban residents due to the lack of access to the micro household data of the National Household Income and Expenditure Survey in our descriptive part. Thus, we can only draw conclusions with a direct descriptive comparison of rural and urban residents’ food consumption with the aggregated data. Second, we concentrate on the analysis of the food purchased externally and do not consider such products which may be unregistered in rural areas due to their own production and consumption. In fact, many rural families in China consume self-produced food. This study does not consider the opportunity costs of obtaining self-produced food due to the lack of relative information. Third, this study only analyzed the main types of food and may not cover all food types, such as soy sauce, vinegar, salt, and many other condiments. The consumption of these items is also necessary for the diet of rural Chinese residents, which may affect the results to some degree. Future studies should consider whole foods including self-produced food, pre-made food, and restaurant food or delivery food, and explore the heterogenous effect of aging on household food consumption between rural and urban residents if needed data are available.

## Data availability statement

The datasets presented in this article are not readily available because this is internal agency data. Requests to access the datasets should be directed to the Fixed Observation Point Management Office of the Research Center for Rural Economy under the Ministry of Agriculture and Rural Affairs of China.

## Author contributions

MG, BW, and WJ involved in conceptualization. JWang and JL conducted the formal analysis and involved in writing the original draft preparation and methodology. JWang involved in software and performed data curation. MG, JWang, JWei, and WJ involved in the writing, reviewing, and editing. JWang and JWei performed the visualization. JWei and JL involved in language. MG performed the supervision and project administration. MG, JWang, and JL involved in funding acquisition. All authors read and agreed to the published version of the manuscript.
