# Supplementary material for: Impact of aging on food consumption in rural China: Implications for dietary upgrading and health improvement
Source: Front Nutr. 2022 Nov 24;9:933343. doi: 10.3389/fnut.2022.933343 (PMC9731807; doi:10.3389/fnut.2022.933343)
Supplement: Supplementary file 1 [file Table_1.pdf]

Table S1. Food classification

Food groups	Food items
Grains	Wheat, wheat flour, dry noodles, wet noodles, bread, buns, instant noodles, rice, corn, corn flour, millet and other cereals, sweet potatoes, potatoes, soybeans and other beans, tofu
Edible oil and fats	Vegetable oil, animal oil
Vegetables and edible mushrooms	Fresh vegetables, leaf stems, fruit vegetables and other fresh vegetables, dried vegetables, dried bamboo shoots, other dried vegetables, seaweed, nori, dried mushrooms, fresh mushrooms
Meat and meat products	Pork, beef, and mutton
Poultry	Chicken, duck, and geese
Aquatic products	Fresh fish (shrimp and crab), fresh shellfish, dried fish, and dried shellfish
Eggs	Egg, duck egg, and goose egg
Milk and dairy products	Fresh milk, milk powder, yogurt, and cheese
Melons and fruits	Fresh fruit and nuts
Sugar	Sugar
